# Fabrication of a Selective and Sensitive Sensor Based on Molecularly Imprinted Polymer/Acetylene Black for the Determination of Azithromycin in Pharmaceuticals and Biological Samples

**DOI:** 10.1371/journal.pone.0147002

**Published:** 2016-01-28

**Authors:** Tingting Zhou, Yun Tao, Hua Jin, Bin Song, Tao Jing, Dan Luo, Yusun Zhou, Yikai Zhou, Yong-Ill Lee, Surong Mei

**Affiliations:** 1 State Key Laboratory of Environment Health (Incubation), Key Laboratory of Environment and Health, Ministry of Education, Key Laboratory of Environment and Health (Wuhan), Ministry of Environmental Protection, School of Public Health, Tongji Medical College, Huazhong University of Science and Technology, #13 Hangkong Road, Wuhan, Hubei, 430030, China; 2 Department of Chemistry, Changwon National University, Changwon, 641–773, Republic of Korea; 3 Central Laboratory, Yanbian University Hospital, Yanji, Jilin, 133000, China; University of Akron, UNITED STATES

## Abstract

A new selective and sensitive sensor based on molecularly imprinted polymer/acetylene black (MIP/AB) was developed for the determination of azithromycin (AZM) in pharmaceuticals and biological samples. The MIP of AZM was synthesized by precipitation polymerization. MIP and AB were then respectively introduced as selective and sensitive elements for the preparation of MIP/AB-modified carbon paste (MIP/ABP) electrode. The performance of the obtained sensor was estimated by cyclic voltammetry (CV) and differential pulse voltammetry (DPV) techniques. Compared with non-molecularly imprinted polymer (NIP) electrodes, NIP/ABP electrodes, and MIP-modified carbon paste electrodes, MIP/ABP electrode exhibited excellent current response toward AZM. The prepared sensor also exhibited good selectivity for AZM in comparison with structurally similar compounds. The effect of electrode composition, extraction parameters, and electrolyte conditions on the current response of the sensor was investigated. Under the optimized conditions, the prepared sensor showed two dynamic linear ranges of 1.0 × 10^−7^ mol L^−1^ to 2.0 × 10^−6^ mol L^−1^ and 2.0 × 10^−6^ mol L^−1^ to 2.0 × 10^−5^ mol L^−1^, with a limit of detection of 1.1 × 10^−8^ mol L^−1^. These predominant properties ensured that the sensor exhibits excellent reliability for detecting AZM in pharmaceuticals and biological fluids without the assistance of any separation techniques. The results were validated by the high-performance liquid chromatography–tandem mass spectrometry method.

## Introduction

**Fig 1:**
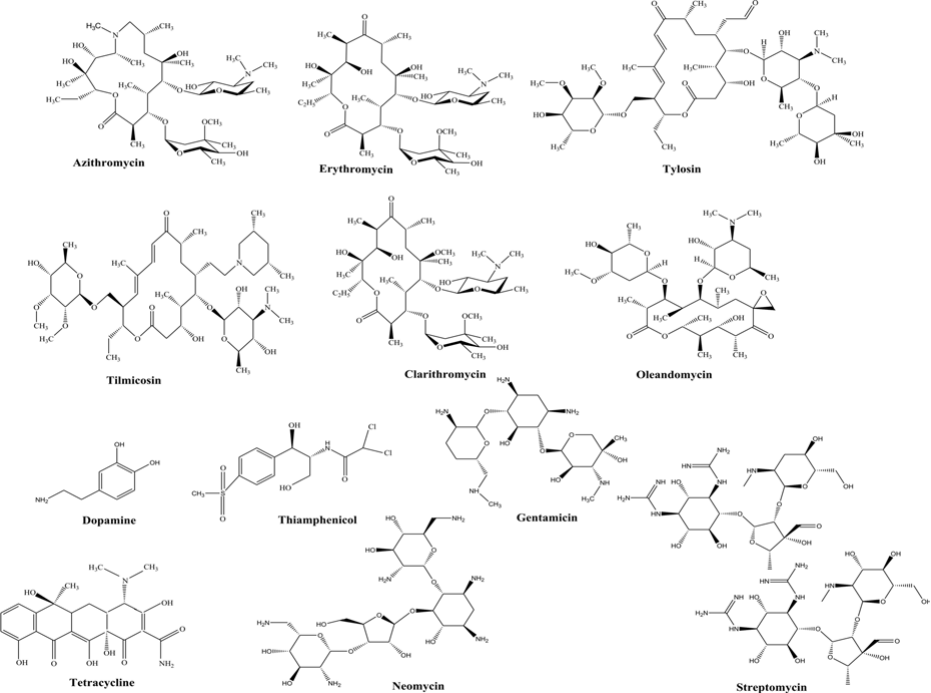
Chemical structures of six MACs and six other compounds tested in this study. The six MACs are AZM, ERY, TM, OLE, TYL, and CLA. The six other compounds are DA, STM, GM, TC, NM, and TAP.

Azithromycin (AZM) (Fig 1) is a macrolide antibiotic (MAC) that is extensively used to treat bacterial infections in human beings, including respiratory, urogenital, pneumococcal pneumonia, and bronchiolitis obliterans [1–3]. However, several side effects will occur when the accumulated dose of AZM exceeds a certain value. Several studies showed that AZM may increase the risk of cardiovascular death and that the interaction between AZM and simvastatin may cause rhabdomyolysis [4,5]. Therefore, the rapid, sensitive, and reliable determination of AZM dosage is of great significance from a health viewpoint.

Various methods have been developed for the detection of AZM, including bioassay [6,7], spectrophotometry [8], liquid chromatography [9–12], high-performance liquid chromatography–tandem mass spectrometry (HPLC-MS/MS) [13,14] and electrochemical methods [15–18]. However, most of these methods are insufficient either in sensitivity, ease of access, or cost effectiveness. Among the aforementioned methods, the electrochemical protocol is preponderant because of its low cost, ease of preparation, fast response, high sensitivity, and real-time detection.

Sensitivity and selectivity are two common requirements in electrochemical analysis. A variety of carbon materials, including graphene [19,20], carbon nanotubes [21,22], mesoporous carbon [23,24], and metal nanoparticles and oxides [25,26] were extensively used to increase the sensitivity of electrochemical determination. A special type of porous carbon material, namely, acetylene black (AB), has recently attracted considerable attention in the field of electrochemical analysis because of its excellent electric conductivity, high accumulation efficiency and large surface area. These properties of AB are beneficial in improving the sensitivity of electrochemical detection. AB has been used in the sensitive determination of paeonol [27], chrysophanol [28], erythromycin (ERY) [29], and glucose [30]. By contrast, biosensors are used as sensing elements to enhance the selectivity of electrochemical analysis to specific enzymes and antibodies [31,32]. However, the activity and stability of enzymes and antibodies are significantly influenced by temperature, organic solvents, and acidic pH, which restrict their applications. Molecularly imprinted polymers (MIPs), which are considered artificial antibodies, contain recognition sites that are complementary in shape, size and chemical functionality to the target molecules. In sensing ability, the utilization of MIP in modifying sensors enables the development of chemical sensors that are comparable with biosensors in sensitivity [33–36]. MIP also has good chemical and physical stability over a wide range of experimental conditions and solvents. Thus, MIPs are extensively used in selective electrochemical analysis.

In this study, we constructed a novel electrochemical sensor for measuring AZM with MIP/AB composite as selective and sensitive sensing elements. ERY, which is a structural analog of AZM, was selected as the template molecule to prepare MIP. To the best of our knowledge, no electrochemical sensors composite have been developed for the detection of AZM based on the MIP/AB composite. The prepared MIP/AB P sensor exhibited excellent selectivity and sensitivity and satisfactory repeatability and stability in AZM analysis. Finally, the sensor was applied successfully for detection of AZM in tablets and biological samples.

## Materials and Methods

### Reagents

AZM, ERY, and tilmicosin (TM) were obtained from Sigma-Aldrich (USA). Oleandomycin (OLE), tylson (TYL), and clarithromycin (CLA) were obtained from Dr. Ehrenstorfer (Germany). Dopamine (DA), streptomycin (STM), gentamicin (GM), tetracycline (TC), neomycin (NM), and thiamphenicol (TAP) were purchased from Probe Biotechnology LLC (China). The chemical molecular structures of these substances are shown in Fig 1. AZM-dispersible tablets (250 mg) were obtained from a local pharmacy. Ethylene glycol dimethacrylate (EGDMA), methacrylic acid (MAA) and 2,2-azobisisobutyronitrile (AIBN) were obtained from Sigma-Aldrich (USA). In order to remove the polymerization inhibitor, MAA and EGDMA were purified by distillation in vacuum under nitrogen protection then stored at 4°C before use. AIBN was recrystallized from hot methanol and dried in a vacuum. AB (purity > 99.99%) was obtained from Strem Chemicals (USA). Paraffin oil and graphite powder were obtained from Sinopharm Chemical Reagent Co., Ltd. (China). Acetonitrile and methanol were purchased from KeMiOu Chemical Reagent Co. (China). All other chemicals were of analytical grade and obtained from Sinopharm Chemical Reagent Co., Ltd. (China). All solutions were freshly prepared with ultrapure water.

### Instruments

The pore structure and surface area of MIPand NIP were detected by using the ASAP 2010 instrument from RMIT Applied Chemistry (Micromeritics, USA) in the Brunauer–Emmett–Teller (BET) nitrogen adsorption and desorption modes after the appropriate degassing and drying of the sample. The surface morphology of MIP was observed by using a field emission scanning electron microscope (Sirion 200, FEI, Holland). Electrochemical data were obtained from the CHI 660C electrochemistry workstations (Shanghai CH Instruments, China). The prepared MIP- or NIP-involved sensor was used as the working electrode. A saturated calomel electrode (SCE) and a platinum wire were employed as the reference and counter electrodes, respectively.

### MIP Synthesis

The MIP synthesis procedure was based on precipitation polymerization according to our previous study [37]. Simplely, the template ERY (1.5 mmol) and functional monomer MAA (6 mmol) were dissolved in 50 mL mixture of methanol/acetonitrile (1/4, v/v) in a 100 mL round-bottomed glass bottle. Thereafter, the mixture was prepolymerized at 40°C for 12 h under stirring. Then, 15 mmol cross-linker (EGDMA) and 40 mg initiator (AIBN) were added after purged with nitrogen for 15 min to remove oxygen, the bottle was sealed and polymerization occurred under magnetic stirring at 60°C for 24 h. The polymer was rinsed with methanol/acetic acid (9/1, v/v) to remove the template until no ERY can be detected by spectrometric measurement (at 210 nm). Finally, the polymer was washed with methanol for several times to remove acetic acid and then dried in oven at 60°C overnight. NIP was prepared similar to the MIP, except that no AZM was added.

### Preparation of the Sensors

The chemically modified carbon paste (CP) electrodes were prepared by completely mixing graphite powder (350 mg), AB (100 mg), MIP or NIP (90 mg), and paraffin oil (60 μL) in a mortar for at least 20 min. Subsequently, the obtained paste was packed into the cavity of the working electrode. The surface of the electrode was polished with butter paper to ensure a reproducible working surface. By scraping out the old surface and replacing the paste, the electrode surface was renewed. Only MIP-modified CP (MIP/CP) or only NIP-modified CP (NIP/CP) was prepared similarly, except for the addition of AB. ABP electrode was prepared under the same conditions, except that MIP or NIP was omitted.

### Measurement of AZM in Pharmaceutical Tablets and Biological Samples

The prepared MIP/ABP sensor was employed to detect AZM in tablets and in serum and urine samples to verify the performance and feasibility of the developed sensor in real samples.

The AZM tablet (containing 250 mg AZM) was ground to a homogeneous fine powder by using a mortar and pestle. The powder was dissolved in methanol, stirred for 10 min, and filtered. The filtered solution was diluted to a certain concentration by using methanol for further analysis.

Drug-free human serum and urine samples were obtained from healthy volunteers, collected, and stored at −20°C until use. Prior to use, the samples were filtered through a 0.45 μm Millipore filter to be clear. The fresh sample solution was prepared by diluting 100 μL of the sample to 10 mL by using phosphate-buffered saline (PBS; 0.1 M, pH 7.0).Three different concentrations of AZM standard solutions were added to the samples before determination for the spiked recovery experiment. The MIP/ABP electrode was then inserted into the sample solution. The target AZM was extracted and preconcentrated on the surface of the electrode for 8 min under a stirring rate of 500 rpm. Subsequently, the electrode was transferred to the electrochemical cell containing 10 mL PBS (0.1 M, pH 7.0) for differential pulse voltammetry (DPV) determination. The DPV method was conducted with a pulse amplitude of 50 mV, pulse width of 40 ms, and scan rate of 20 mV s^−1^ over the potential range of 0.4 V to 1.0 V.

### Ethics Statement

This study was conducted on the basis of the Declaration of Helsinki and approved by the Institutional Review Board of Tongji Medical College. Before the urine and blood samples were obtained, the volunteers were required to sign a written informed consent after receiving a detailed explanation of the study. The volunteer consents were recorded in a file.

## Results and Discussion

### Characterization of MIP

In this study, ERY, which is a structural analog of AZM, was selected as a dummy template to prevent the leakage of residual template molecules and prevent the inaccurate determination of the AZM trace level [38]. According to our previous study, the prepared dummy MIP could selectively recognize the target AZM [37].

The static adsorption capacities of MIP and NIP for AZM were determined in various concentrations of AZM ranging from 0.5 g L^−1^ to 4.0 g L^−1^. The adsorption capacity (*Q*, mg g^−1^) was calculated on the basis of the difference in AZM concentrations before and after adsorption by the polymer by using a constant volume of acetonitrile and a precise weight of MIP or NIP in accordance with the following equation:

$$Q=\left(C_{0}-C\right)V/M,$$

in which *C*_0_ is the initial AZM concentration (mg L^−1^), *C* is the AZM concentration after adsorption, *V* is the volume of acetonitrile (mL), and *M* is the weight of the polymer (g). As shown in S1 Fig, the adsorption capacities of MIP and NIP increased with the increasing of AZM concentrations. The weak adsorption of AZM on NIP was mainly due to the nonspecific interaction between AZM and NIP.

The porosity of MIP and NIP was investigated by nitrogen adsorption–desorption experiment. According to the test results, the BET surface area was 118.98 m^2^ g^−1^ for MIP and 30.45 m^2^ g^−1^ for NIP. The pore volume of MIP and NIP was 0.57 and 0.08 cm^3^ g^−1^, respectively. The results showed that MIP was more porous than NIP, thus partly explaining why MIP had a higher adsorption capacity than NIP.

**Fig 2:**
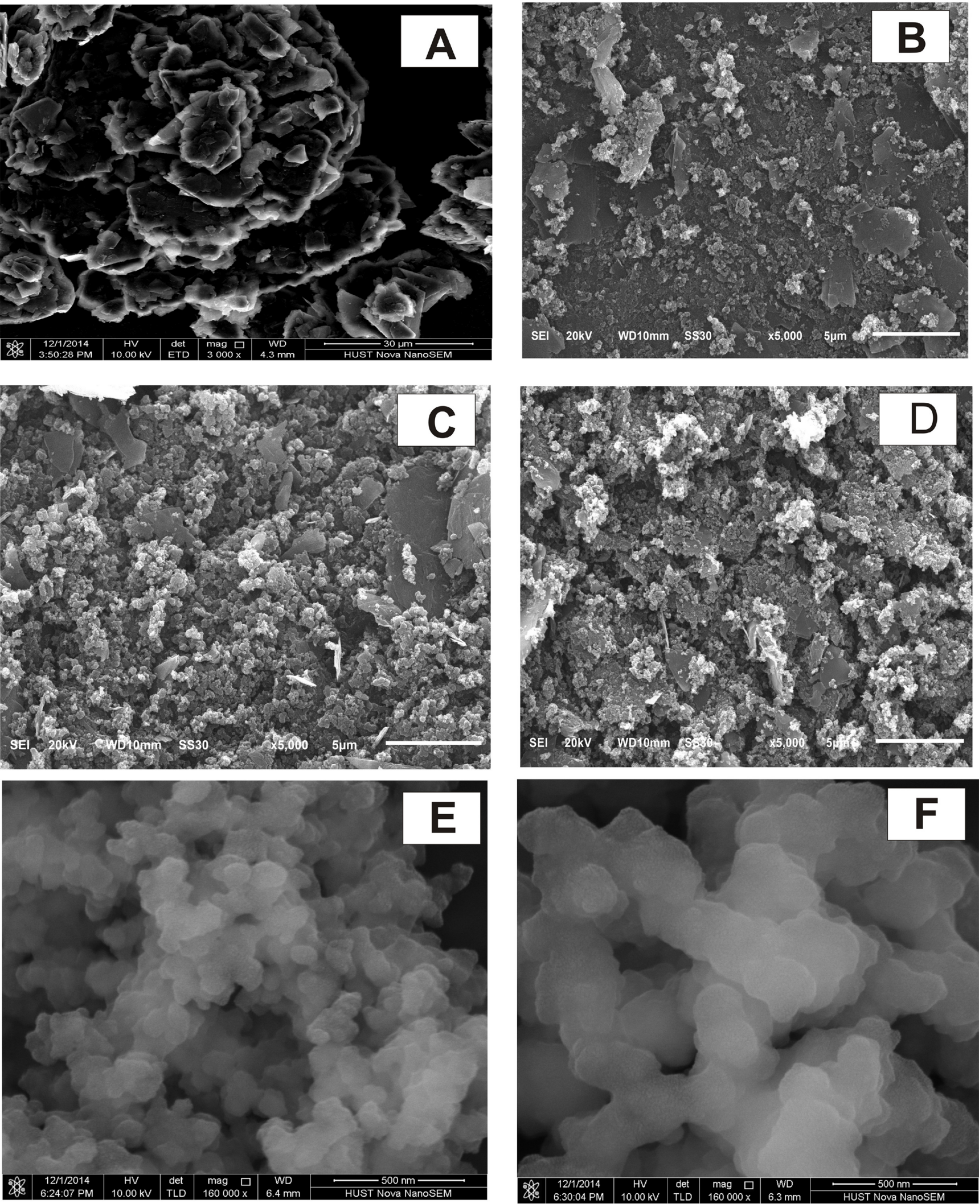
The SEM images of various electrodes, MIP and NIP. CP (A), ABP (B), MIP/ABP (C), NIP/ABP (D) electrodes, MIP (E) and NIP (F).

The surface morphologies of MIP/ABP electrode, NIP/ABP electrode, ABP electrode, and CP electrode were investigated by scanning electron microscopy. The surfaces of the MIP/ABP electrode (Fig 2C) and NIP/ABP electrode (Fig 2D) were more difference with that of the CP electrode (Fig 2A) and ABP electrode (Fig 2B). As such, we conclude that MIP or NIP was appeared on the surface area of the sensor. MIP (Fig 2E) is also more porous than NIP (Fig 2F), thus partly explaining why MIP has a larger adsorption capacity for AZM than NIP.

### Electrochemical Behavior of AZM

**Fig 3:**
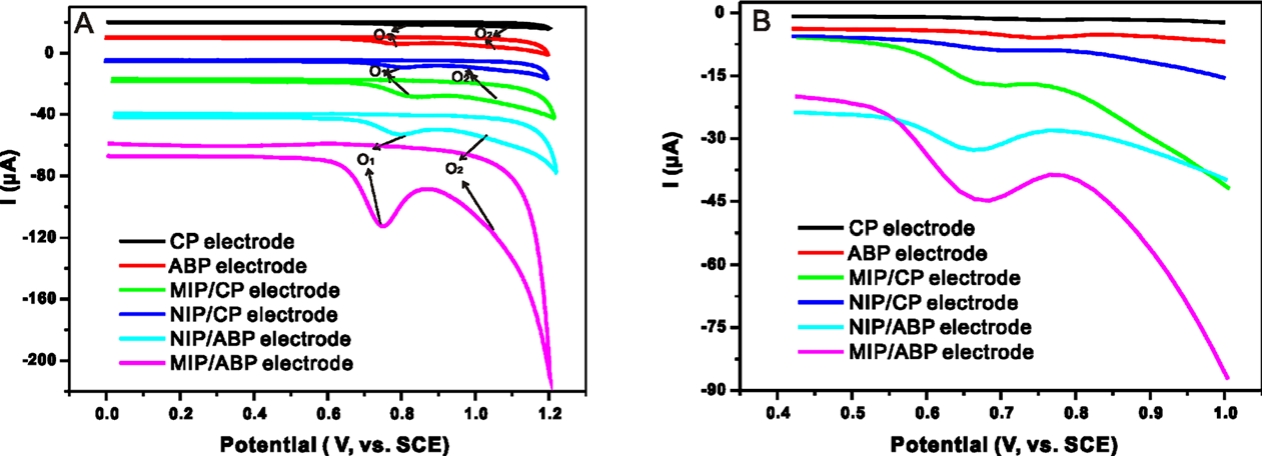
Cyclic voltammograms (A) and differential pulse voltammograms (B) of AZM on CP, ABP, MIP/CP, NIP/CP, MIP/ABP and NIP/ABP electrode in PBS (0.1 M, pH 7.0). Conditions: (A) The concentration of AZM is 1 × 10^−5^ mol L^−1^, potential scan range: 0.4 V–1.0 V, scan rate: 100 mV s^−1^; (B) The concentration of AZM is 2 × 10^−6^ mol L^−1^, scan rate: 20 mV s^−1^, pulse amplitude: 50 mV, pulse width: 40 ms.

In the primary experiments, the electrochemical behavior of AZM on CP, ABP, MIP/CP, NIP/CP, NIP/ABP, and MIP/ABP electrodes were investigated by CV in PBS (0.1 M, pH 7.0). The resulting voltammograms (Fig 3A) showed two anodic oxidation peaks (O_1_ and O_2_) at 0.8 and1.0 V.However, no peak was observed in the opposite direction. This finding indicated that the electrochemical reaction of AZM was an irreversible process. Among the different functional groups of AZM, the most easily oxidized group is the dialkylamine group [39]. Dialkylamines oxidize to form a radical cation by the loss of one electron. The peak O_1_ may be attributed to the form of an aminium cation radical from one of the nitrogen atoms by losing an electron. The peak O_2_ may be resulted from the oxidation of the product of the aminium cation radical [17].

The DPV method was employed to investigate the recognition ability of the prepared sensor further. As shown in Fig 3B, the current response of AZM on the MIP/CP and MIP/ABP electrodes was higher than that on the NIP/CP and NIP/ABP electrodes. This indicated that the electrode modified with MIP can intensively absorb AZM from the aqueous solution compared with the electrode modified with NIP because of the selective cavities existing in MIP during the polymerization steps. The DPV signal of the MIP/ABP electrode was also higher than that of the MIP/CP electrode. This indicated that the addition of AB enhanced the sensitivity of the sensor.

### Optimization of the Experimental Parameters for the Developed MIP/ABP Sensor

#### Effect of Electrode Composition on MIP/ABP Electrode Performance

**Fig 4:**
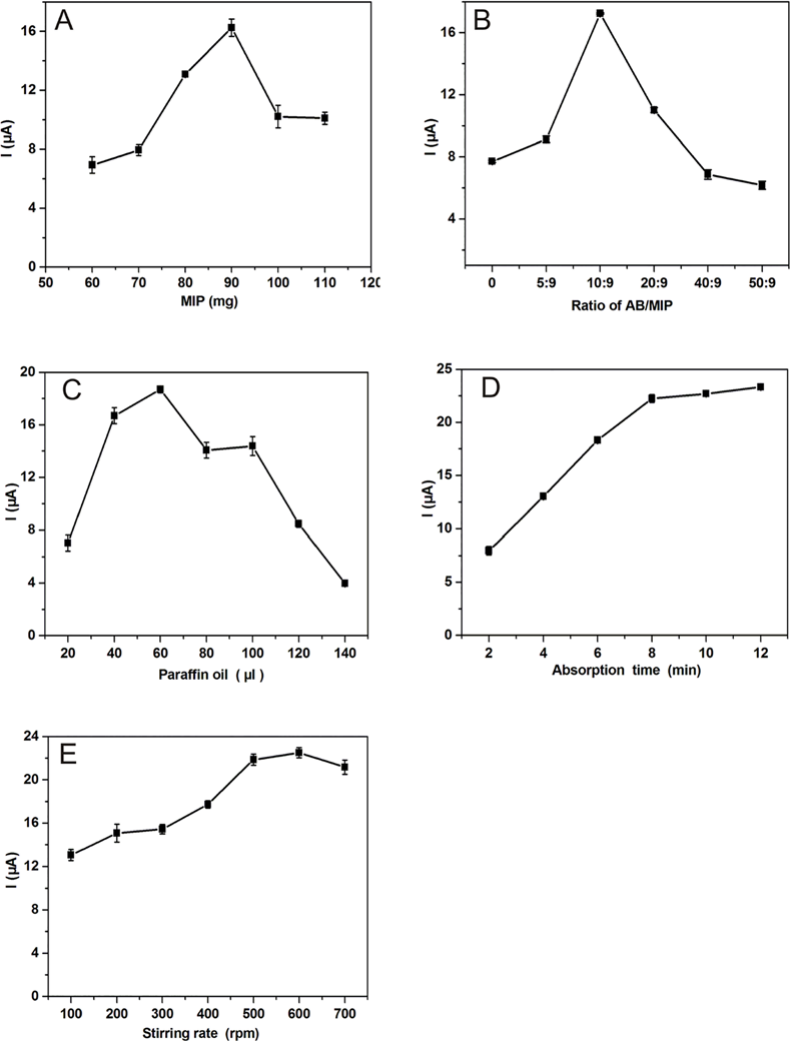
Optimization of different conditions affecting the current response of AZM, including the amount of MIP (A), the ratio of AB/MIP (B), the amount of paraffin oil (C), incubation time (D), and stirring rate (E). The other conditions are the same as that in Fig 3B.

The amount of MIP, paraffin oil and the ratio of AB/MIP were optimized with one variable at a time to determine the best composition of the MIP/ABP electrode in the fixed conditions of extraction and determination. Fig 4A shows that the maximum response of the prepared sensor was achieved when the amount of MIP was 90 mg. High amounts of MIP in the MIP/ABP electrode can increase the response of the sensor because of more recognition sites on the electrode surface. However, when the amount of MIP was more than 90 mg, a decrease in sensor response was observed because of the decrease in conductivity on the electrode surface. As the sensitive and selective sensing elements for the sensor, the ratio of AB/MIP is important to the performance of the sensor. Fig 4B shows the effect of the ratio of AB/MIP on the sensor performance. The peak current of AZM decreased when the ratio of AB/MIP was lower than 10:9 because the decrease in AB on the electrode surface led to the decrease in sensor conductivity. When the ratio of AB/MIP was higher than 10:9, the peak current of AZM also decreased because the decrease in MIP resulted in the reduction of recognition sites on the surface of the electrode. Therefore, considering sensitivity and selectivity, 10:9 was selected as the best ratio of AB/MIP. The optimum amount of paraffin oil was required for the preparation of the MIP/ABP electrode. As shown in Fig 4C, 60 μL paraffin oil was the best option for preparing the MIP/ABP electrode. In the presence of insufficient amount of paraffin oil, the physical property of the electrode was not able to provide a durable and repeatable electrode surface. However, a high amount of paraffin oil in the electrode led to a decrease in current response because paraffin oil is an insulating material, thus decreasing the conductivity of the electrode surface.

#### Optimization of Extraction Conditions

The influence of extraction time on the response of the sensor was examined over a time period of 2 min to 12 min. As shown in Fig 4D, the increase of the extraction time led to an intensive increase in the current response up to approximately 8 min. The increase rate of current response was slow down with the increase of extraction time. Thus, 8 min was selected as the optimum extraction time. The effect of stirring rate on AZM extraction was also investigated in the range of 100 to 600 rpm. The results are shown in Fig 4E. The current remained constant at stirring rates higher than 500 rpm. Therefore, the stirring rate of 500 rpm was selected for subsequent use.

**Fig 5:**
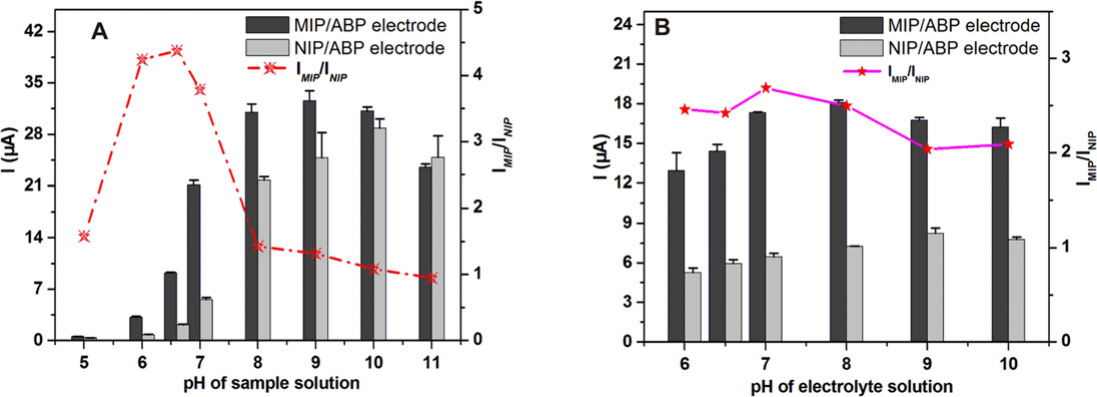
Effects of the pH value of the sample solution (A) and electrolyte solution (B) on the current responses of 2 × 10^−6^ mol L^−1^ AZM at MIP/ABP electrode. The other conditions are the same with that in Fig 3B.

The influence of the pH of the sample solution on the oxidation peak current of AZM was investigated over the pH range of 5.0 to 11.0. As shown in Fig 5A, the current response of the MIP/ABP electrode (*I*_MIP_) initially increased from pH 5.0 to 9.0 and then decreased from 9.0 to 11.0. Similarly, the current response of the NIP/ABP electrode (*I*_NIP_) initially increased from pH 6.0 to 10.0 and then decreased from pH 10.0 to 11.0. However, under the same pH condition, *I*_MIP_/*I*_NIP_ initially increased with the increase of pH from 5.0 to 6.5 and then decreased with the increase of pH from 6.5 to 11.0. *I*_MIP_/*I*_NIP_ refers to the separation factor and reflects the selectivity of the developed sensor. As can be seen in Fig 5A, the variations of *I*_MIP_, *I*_NIP_, and *I*_MIP_/*I*_NIP_ are significantly different. In addition to the complementary interaction, the difference was due to the interplay of the hydrophobic effect between target molecules and polymers. The hydrophobic effect is an applied force to transfer nonpolar molecular from water to a nonpolar medium [40]. Therefore, the target AZM, with evident hydrophobic structures was prone to diffuse onto the hydrophobic surfaces of the prepared electrode surface with MIP and recognized by the specific binding sites. When the pH value increased, hydrogen bond between molecules was weakened, in this case, hydrophobic effect will play an significant role in the molecule recognition [41]. Therefore, the specific hydrogen binding between AZM and MIP was weakened with the increasing pH of the sample solution. The weakened interaction consequently led to the decrease in *I*_MIP_/*I*_NIP_. Although *I*_MIP_/*I*_NIP_ is largest when the pH was 6.5, the current response of the sensor was much higher when the pH was 7.0. Considering sensitivity and selectivity, pH 7.0 was selected as the appropriate pH of the sample solution for further studies.

#### Optimization parameters of Electrochemical Measurement

The effect of the pH of the electrolyte solution on the peak current of AZM was investigated by varying the pH in the range of 6.0 to 10.0. As shown in Fig 5B, the peak current of the MIP/ABP electrode increased with the increasing pH value from 6.0 to 7.0 and then decreased with a further increase of pH value range from 7.0 to 10.0. *I*_MIP_/*I*_NIP_ was changed similarly. Therefore, PBS with pH of 7.0 was selected as the optimum electrolyte solution.

In the case of DPV measurement, the main parameters, pulse amplitude, pulse width, and scan rate, were surveyed over the ranges of 10 to 300 mV, 10 to 100 ms, and 5to 30 mV s^−1^, respectively. The best sensitivity and a well-shaped wave with a relatively narrow peak width were obtained when 50 mV, 40 ms, and 20 mV s^−1^ were selected for pulse amplitude, pulse width, and scan rate.

### The Selectivity of the Developed Electrochemical Sensor with MIP/AB as Sensing Element

**Fig 6:**
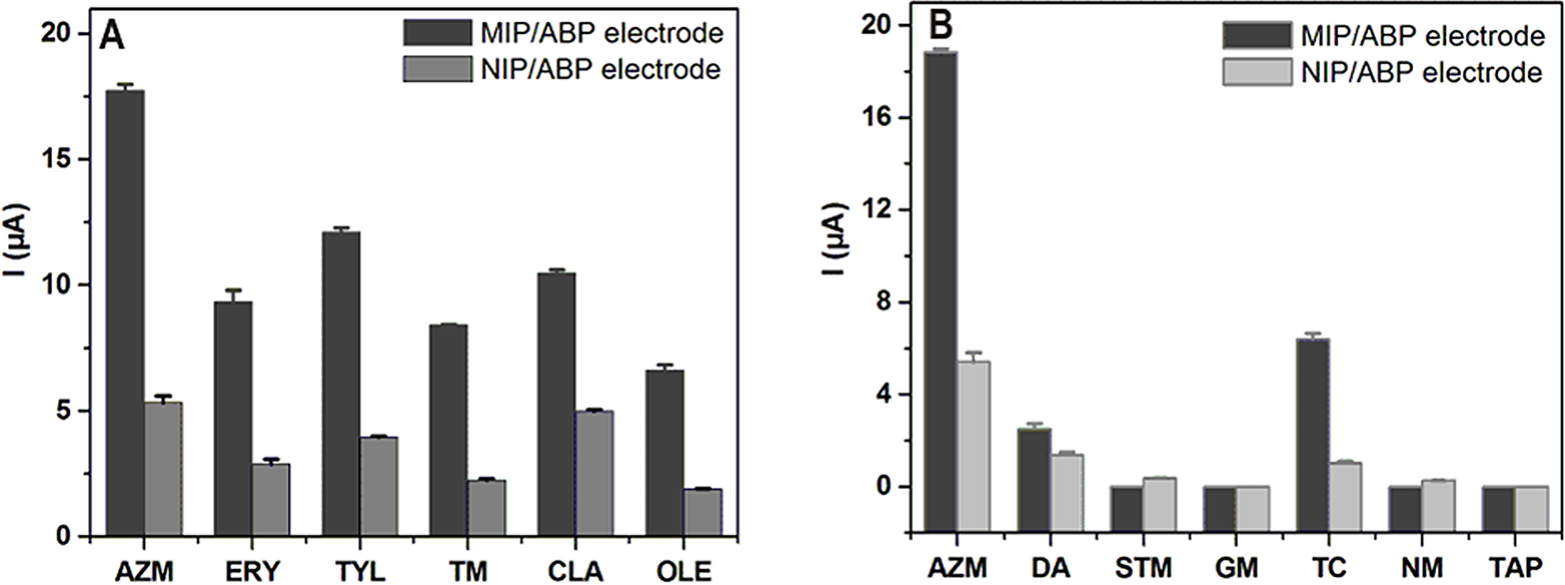
Current responses of 2 × 10^−6^ mol L^−1^AZM and other five MACs obtained at the MIP/ABP and NIP/ABP electrodes (A). Current responses of 2 × 10^−6^ mol L^−1^ AZM and 2 × 10^−4^ mol L^−1^ other six compounds obtained at the MIP/ABP and NIP/ABP electrodes (B). The other conditions are the same as that in Fig 3B.

The selectivity of the designed MIP/ABP sensor was evaluated by inserting the prepared MIP/ABP electrode into the solutions of AZM and other MACs, such as TM, OLE, TYL, CLA, and ERY. Fig 6A shows the DPV responses of the MIP/ABP and NIP/ABP electrodes to the tested compounds. The response of the MIP/ABP electrode to AZM was higher than that of other MACs and the response of the MIP/ABP electrode to the six MACs was higher than that of the NIP/ABP electrode. As shown in Fig 1, compared with other MACs, AZM has one more dialkylamine group, thus enhancing the hydrogen-bonding affinity between AZM and the selective recognition sites on MIP. What is more, the dialkylamine group is electrochemically active, thus resulting in a higher peak current of AZM during electrochemical analysis. Therefore, the prepared sensor shows excellent selectivity toward AZM. The response of the prepared sensor to other antibiotics and electrochemically active compounds, such as STM, GM, TC, NM, TAP, and DA, was significantly small. As shown in Fig 6B, the current peak of AZM was much higher than that of other compounds even when the concentration of AZM is 100-fold lower. As shown in Fig 1, the structures of STM, GM, TC, NM, TAP, and DA are significantly different from that of the template. Thus, STM, GM, TC, NM, TAP, and DA can not be easily recognized and adsorbed into the cavities of MIP.

### Interference Studies

The effect of several organic and inorganic compounds on the detection of AZM was investigated by adding the interfering compounds in the biological samples to the 2 × 10^−6^ mol L^−1^ AZM solution under the optimum measurement conditions. The maximum concentration of a substance when it yielded a relative error of 5% was considered as the tolerance limit. And the mole ratio of the tolerance limit of the interferent and the concentration of AZM is the interferent level. The results shown in Table 1 indicated that the prepared sensor was unaffected by different organic and inorganic substances, even when their concentrations were higher than that of AZM.

**Table 1:** Interference levels of several tested substances in the determination of 2 × 10^−6^ mol L^−1^ AZM by the developed sensor.

Substances	Interference level (mole ratio)
Cu^2+^, Fe^2+^, Fe^3+^, Zn^2+^, Mg^2+^, TAP, chloramphenicol, and uric acid	100
Al^3+^ and Ca^2+^	200
GM, urea, glycine, l-glutamic acid, and creatinine	500
K^+^, Cl^−^, Na^+^, H_2_PO^4−^, HPO_4_^2−^, vitamin C, glucose, and sucrose	1000

### Analytical Characterization

**Fig 7:**
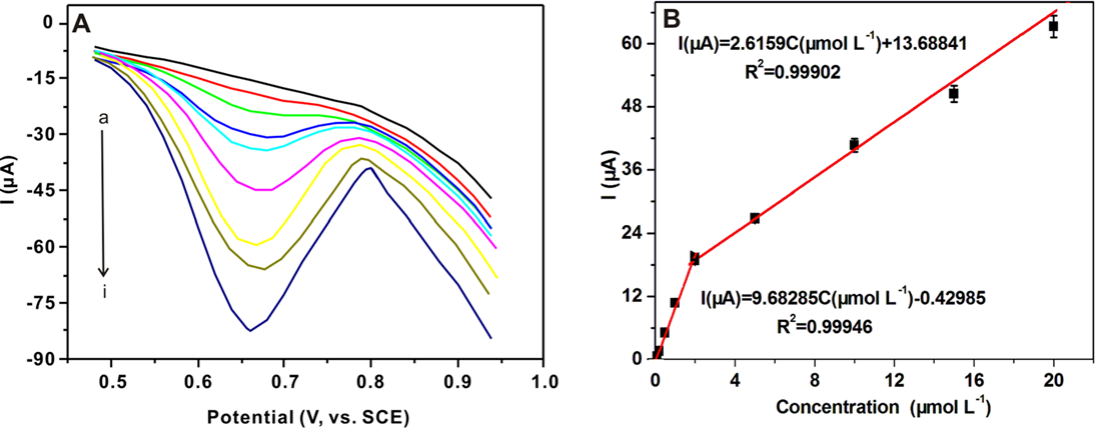
DPV curves (A) of the MIP/ABP electrode in different concentrations of AZM (from a to i): 0.1, 0.2, 0.5, 1, 2, 5, 10, 15 and 20 μM. Calibration curve (B) of AZM under the optimized conditions.

The calibration curve was plotted under the optimal conditions and parameters. As shown in Fig 7A, the response of AZM on the MIP/ABP electrode was enhanced with increasing concentrations from 1.0 × 10^−7^ mol L^−1^ to 2.0 × 10^−5^ mol L^−1^. As shown in Fig 7B, two linear relationships were presented as *I* (μA) = 9.68 *C*_*AZM*_ (μmol L^−1^) − 0.43 (*r*^2^ = 0.9995) in the range of 1.0 × 10^−7^ mol L^−1^ to 2.0 × 10^−6^ mol L^−1^ and *I* (μA) = 2.62 *C*_*AZM*_ (μmol L^−1^) + 13.69 (*r*^2^ = 0.9990) in the range of 2.0 × 10^−6^ mol L^−1^ to 2.0 × 10^−5^ mol L^−1^, where *C*_*AZM*_ denotes the AZM concentration. The limit of detection (LOD) of the method was evaluated according to the 3s/b criteria, in which s is the standard deviation of the blank and b is the slope of the linear calibration curve. Finally, the LOD was 1.1 × 10^−8^ mol L^−1^.

The stability and reproducibility of the proposed sensor were also investigated. No significant performance change was observed with a relative standard deviation (RSD) of 3.8% after the electrode underwent three assays every day for one week. Acceptable reproducibility was observed, with the RSD of 4.26% for eight parallel detections. Table 2 shows the comparisons of different types of modified electrodes for the determination of AZM. The developed sensor in this study exhibited superior selectivity because no electrodes in previously published studies were modified by using selective elements. The LOD of our method was the lowest, thus indicating that the developed sensor exhibited superior sensitivity.

**Table 2:** Comparison of the presented MIP/ABP electrode with other reported electrodes for AZM determination.

Type of electrode	Linear range (μmol L^−1^)	LOD (μmol L^−1^)	Real samples	Reference
GO-MWCNT/GCE^a^	0.1–10	0.07	Pharmaceuticals	[15]
Gr-IL/GCE^b^	0.65–38.0	0.25	Tablets	[16]
Bare GCE^c^	1.34–20.2	0.093	–	[17]
MgCr_2_O_4_-MWCNT/GCE^d^	0.25–10.0	0.07	Pharmaceuticals, plasma, and urine	[18]
MIP/ABP electrode	0.1–20.0	0.011	Serum, urine, and tablets	This study

^a^Graphene oxide–multiwall carbon nanotube/glassy carbon electrode.

^b^Graphene-1-butyl-3-methylimidazolium hexafluorophosphate/glassy carbon electrode.

^c^Glassy carbon electrode.

^d^MgCr_2_O_4_ nanoparticle–multiwall carbon nanotube/glassy carbon electrode.

### Analytical Application

The developed MIP/ABP sensor was applied to analyze AZM in tablets and biological samples. The HPLC-MS/MS method [14] was applied to detect AZM in real samples to verify the performance of the proposed method statistically. The results are shown in Table 3. A good agreement between the results obtained by our proposed electrochemical method and the HPLC-MS/MS method was observed. These results evidently indicated that the prepared sensor could be reliable and effective for the determination of AZM in real samples.

**Table 3:** Detection of AZM in real samples by the developed electrochemical method (*n* = 3) and HPLC-MS/MS method (*n* = 3).

Sample			Electrochemical			LC-MS/MS	
	Added (μmol/L)	Found (μmol/L)	Recovery (%)	RSD (%)	Found (μmol/L)	Recovery (%)	RSD (%)
Urine 1	0.5	0.495	99	1.9	0.483	96.6	2.1
	2	1.807	90.4	1.6	2.013	100.7	1.9
	5	5.288	105.6	3.8	4.907	98.1	4.2
Urine 2	0.5	0.521	105.8	4.6	0.493	98.6	3.6
	2	1.818	90.9	2.8	1.962	98.1	3.2
	5	5.182	103.6	3.4	35.612	104.5	4.1
Urine 3	0.5	0.499	99.8	5.1	0.485	97	2.9
	2	1.812	90.6	1.7	2.053	102.7	1.8
	5	5.147	102.9	5.0	4.924	98.5	5.3
Serum 1	0.5	0.499	99.8	4.2	0.511	102.2	2.7
	2	1.885	94.3	2.6	1.933	96.7	3.1
	5	5.372	107.4	2.1	4.961	99.2	4.3
Serum 2	0.5	0.516	103.2	3.9	0.508	101.6	3.7
	2	2.039	102	2.7	2.044	102.2	2.6
	5	5.417	108.3	0.74	5.093	101.9	1.3
Serum 3	0.5	0.513	102.6	2.6	0.492	98.4	0.84
	2	1.869	93.5	1.1	1.987	99.4	2.4
	5	5.129	102.6	1.3	5.053	101.1	4.4
Tablet 1	0.25*	0.234	93.6	0.42	0.233	93.2	0.11
Tablet 2	0.25*	0.248	99.2	0.12	0.226	90.4	0.12
Tablet 3	0.25*	0.233	93.2	0.41	0.242	96.8	0.32

*Labeled content (g) in AZM tablet.

## Conclusion

In summary, a novel and facile electrochemical sensor was fabricated for the determination of AZM in pharmaceuticals and biological samples. The resulting sensor exhibited outstanding selectivity and sensitivity in AZM detection because of the effective integration of MIP and AB as sensing elements. Compared with previously reported electrochemical sensors, the designed sensor exhibited better selectivity and lower LOD. The proposed sensor was cost effective and exhibited satisfactory applicability. As such, the proposed sensor could be directly applied to the determination of AZM in complex matrices without prior sample preparation or separation.

## Supporting Information

S1 Fig Static adsorption curves of MIP and NIP for AZM. (DOCX)
