# Supplementary material for: Fabrication of a Selective and Sensitive Sensor Based on Molecularly Imprinted Polymer/Acetylene Black for the Determination of Azithromycin in Pharmaceuticals and Biological Samples
Source: PLoS One. 2016 Jan 28;11(1):e0147002. doi: 10.1371/journal.pone.0147002 (PMC4731201; doi:10.1371/journal.pone.0147002)
Supplement: S1 Fig — (DOCX) [file pone.0147002.s001.docx]

S1 Fig. Static adsorption curves of MIP and NIP for AZM.
